# Probing human sperm metabolism using ^13^C-magnetic resonance spectroscopy

**DOI:** 10.1093/molehr/gay046

**Published:** 2018-11-03

**Authors:** S J Calvert, S Reynolds, M N Paley, S J Walters, A A Pacey

**Affiliations:** 1 Academic Unit of Reproductive & Developmental Medicine, Department of Oncology and Metabolism, University of Sheffield, Level 4, The Jessop Wing, Tree Root Walk, Sheffield, UK; 2 Academic Unit of Radiology, Department of Immunity, Infection and Cardiovascular Disease, University of Sheffield, Sheffield, UK; 3 School of Health Related Research, University of Sheffield, Regent Court, 30 Regent Street, Sheffield, UK

**Keywords:** magnetic resonance spectroscopy, metabolism, glycolysis, Krebs cycle, human sperm

## Abstract

**STUDY QUESTION:**

Can ^13^C-Magnetic Resonance Spectroscopy (MRS) of selected metabolites provide useful information about human sperm metabolism and how glycolysis or oxidative phosphorylation are used by different sperm populations?

**SUMMARY ANSWER:**

Sperm populations, prepared by density gradient centrifugation (DGC) and incubated with either ^13^C_u_-glucose, ^13^C_u_-fructose or ^13^C_1_-pyruvate, showed consistent evidence of metabolism generating principally lactate and more intermittently bicarbonate, and significantly more lactate was produced from ^13^C_u_-glucose by vital or motile sperm recovered from the 40/80% interface compared to those from the pellet, which could not be accounted for by differences in the non-sperm cells present.

**WHAT IS KNOWN ALREADY:**

Previous studies have focused on CO_2_ or other specific metabolite production by human sperm and there remains considerable debate about whether glycolysis and/or oxidative phosphorylation is the more important pathway for ATP production in sperm.

**STUDY DESIGN, SIZE, DURATION:**

Sperm populations were prepared by DGC and subjected to ^13^C-MRS to answer the following questions. (i) Is it possible to detect human sperm metabolism of ^13^C substrates implicated in energy generation? (ii) What are the kinetics of such reactions? (iii) Do different sperm populations (e.g. ‘80%’ pellet sperm and ‘40%’ interface sperm) utilise substrates in the same way? Semen samples from 97 men were used in these experiments; 52 were used in parallel for aims (i) and (ii) and 45 were used for aim (iii).

**PARTICIPANTS/MATERIALS, SETTING, METHODS:**

Sperm populations were prepared from ejaculates of healthy men using a Percoll/Phosphate Buffered Saline (PBS) DGC and then incubated with a range of ^13^C-labelled substrates (^13^C_u_-glucose, ^13^C_u_-fructose, ^13^C_1_-pyruvate, ^13^C_1_-butyrate, ^13^C_3_-lactate, ^13^C_2,4_-D-3-hydroxybutyrate, ^13^C_5_-l-glutamate, ^13^C_1,2_-glycine or ^13^C_u_-galactose) along with penicillin/streptomycin antibiotic at 37°C for 4 h, 24 h or over 48 h for an estimated rate constant. Sperm concentration, vitality and motility were measured and, for a subset of experiments, non-sperm cell concentration was determined. A 9.4 T magnetic resonance spectrometer was used to acquire 1D ^13^C, inverse gated ^1^H decoupled, MRS spectra. Spectrum processing was carried out using spectrometer software and Matlab scripts to determine peak integrals for each spectrum.

**MAIN RESULTS AND THE ROLE OF CHANCE:**

^13^C_u_-glucose, ^13^C_u_-fructose and ^13^C_1_-pyruvate were consistently converted into lactate and, to a lesser extent, bicarbonate. There was a significant correlation between sperm concentration and lactate peak size for ^13^C_u_-glucose and ^13^C_u_-fructose, which was not observed for ^13^C_1_-pyruvate. The lactate peak did not correlate with the non-sperm cell concentration up to 6.9 × 10^6^/ml. The concentration of ^13^C_u_-glucose, ^13^C_u_-fructose or ^13^C_1_-pyruvate (1.8, 3.6, 7.2 or 14.4 mM) had no influence on the size of the observed lactate peak over a 4 h incubation. The rate of conversion of ^13^C_1_-pyruvate to lactate was approximately three times faster than for ^13^C_u_-glucose or ^13^C_u_-fructose which were not significantly different from each other. After incubating for 4 h, the utilisation of ^13^C_u_-glucose, ^13^C_u_-fructose or ^13^C_1_-pyruvate by sperm from the ‘40%’ interface of the DGC was no different from those from the pellet when normalised to total sperm concentration. However, after normalising by either the vital or motile sperm concentration, there was a significant increase in conversion of ^13^C_u_-glucose to lactate by ‘40%’ interface sperm compared to pellet sperm (Vital = 3.3 ± 0.30 × 10^6^ vs 2.0 ± 0.21 × 10^6^; *P* = 0.0049; Motile = 7.0 ± 0.75 × 10^6^ vs 4.8 ± 0.13 × 10^6^; *P* = 0.0032. Mann–Whitney test *P* < 0.0055 taken as statistically significant). No significant differences were observed for ^13^C_u_-fructose or ^13^C_1_-pyruvate.

**LARGE SCALE DATA:**

Not applicable.

**LIMITATIONS, REASONS FOR CAUTION:**

Only ^13^C labelled metabolites that accumulate to a sufficiently high concentration can be observed by ^13^C MRS. For this reason, intermediary molecules in the metabolic chain are difficult to observe without trapping the molecule at a particular step using inhibitors. Non-sperm cell concentration was typical of the general population and no link was found between these cells and the magnitude of the ^13^C-lactate peak. However, it is possible that higher concentrations than the maximum observed (6.9 × 10^6^/ml) may contribute to exogenous substrate metabolism in other experiments.

**WIDER IMPLICATIONS OF THE FINDINGS:**

^13^C-MRS can provide information on the underlying metabolism of multiple pathways in live sperm. Dysfunction in sperm metabolism, as a result of either impaired enzymes of lack of metabolisable substrate, could be detected in sperm by a non-destructive assay, potentially offering new treatment options to improve overall sperm quality and outcomes for reproduction.

**STUDY FUNDING AND COMPETING INTERESTS:**

This work was supported by the Medical Research Council Grant MR/M010473/1. The authors declare no conflicts of interest.

## Introduction

Poor sperm quality significantly contributes to cases of infertility within couples (Pacey, 2009), yet many basic aspects of sperm physiology remain unknown (Barratt *et al.*, 2018). One important and unanswered question is how human sperm generate the ATP (adenosine triphosphate) necessary to sustain motility and undergo the metabolically demanding processes of capacitation and hyperactivation (Suarez and Pacey, 2006). Furthermore, such information may be useful both to help understand the molecular causes of poor sperm motility (e.g. asthenozoospermia) and to provide insights into targets for novel agents to enhance sperm motility.

After more than 50 years of research, it is now clear that human sperm can produce ATP through the metabolic processes of glycolysis and/or oxidative phosphorylation (du Plessis *et al.*, 2015). This has been examined using a variety of experimental approaches over the years, including: (i) the measurement of oxygen consumption of washed sperm either in the presence (Ford and Harrison, 1981) or absence (Peterson and Freund, 1970) of metabolic inhibitors; (ii) incubation with ^14^C radiolabelled substrates (Murdoch and White, 1968; Ford and Harrison, 1981); (iii) the measurement of ADP and ATP in semen samples with different phenotypes (Vigue *et al.*, 1992); and (iv) the use of proteomics to identify new metabolic enzymes and pathways (Amaral *et al.*, 2014). However, there remains considerable debate about whether glycolysis and/or oxidative phosphorylation is more important for the various aspects of human sperm function during their post-ejaculatory life (Ruiz-Pesini *et al.*, 2007; du Plessis *et al.*, 2015).

In a recent paper, we used ^1^H Magnetic Resonance Spectroscopy (MRS) to examine the endogenous metabolome of live human sperm isolated from semen using 40/80% Density Gradient Centrifugation (DGC) (Reynolds *et al.*, 2017a). This showed that several metabolite peaks, including those associated with lactate, could be used to discriminate sperm recovered from the pellet (‘80%’ sperm) from those recovered from the ‘40%’ interface. As ‘80%’ sperm typically have better motility, this suggested that there may be important metabolic differences between these two sperm populations with respect to their utilisation of the pathways of glycolysis and oxidative phosphorylation.

^13^C-MRS has been used to examine metabolic pathways in other cell types (Buescher *et al.*, 2015) including metabolic regulation in cancer cells (Shestov *et al.*, 2016). Using ^13^C labelled substrates provides three advantages. Firstly, they are metabolised the same as those found within human physiology and their ^13^C-MRS spectra are greatly simplified compared to ^1^H MRS, displaying known peaks for the source substrate and those peaks having arisen from cellular metabolism. Secondly, particular metabolic pathways can be identified through strategic placement of the ^13^C label (Buescher *et al.*, 2015). Alternative or multiple pathways can be assayed through varying ^13^C labelling patterns, even if the end product is the same (Bruntz *et al.*, 2017). Finally, the cells under study remain viable throughout the experiment and therefore can be measured at multiple time points (Reynolds *et al.*, 2017b).

As many aspects of sperm metabolism remain unknown and ^13^C-MRS can provide insights into metabolism in live cells, we reasoned that this combination would be able to further elucidate the metabolic pathways used by live human sperm. In this paper, we use ^13^C-MRS to investigate three questions: (i) Is it possible to detect human sperm metabolism of ^13^C substrates implicated in energy generation? (ii) What are the kinetics of such reactions? (iii) Do different sperm populations (e.g. ‘80%’ and ‘40%’ sperm) utilise substrates in the same way?

## Materials and Methods

### Semen donation and analysis

Semen samples were obtained from men attending the Andrology Laboratory (Jessop Wing, Sheffield, UK) for semen analysis (approved by the North of Scotland Research Ethics Committee (16/NS/0009) on 17/02/16). Informed consent was obtained from each man to use their ejaculates in this project and semen samples were produced after at least 2 days of sexual abstinence. Each ejaculate was collected into a sterile plastic container (Sarstedt, Leicester, UK) and examined according to World Health Organisation (2010) methods within one hour of production. Samples selected for experiments contained at least a total of 25 × 10^6^ sperm and 40% progressively motile sperm, as these contain sufficient sperm of normal motility to complete sample preparation.

### Sperm preparation techniques

**Figure 1:**
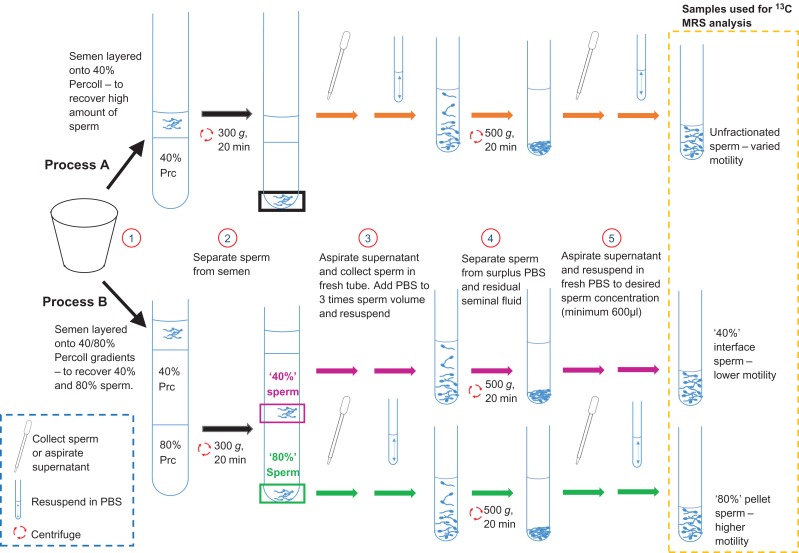
Sperm washing methods used to remove seminal plasma. Process (A) was used to yield higher concentration of sperm for aims (i) and (ii). Process (B) was used to fractionate sperm into higher average motility, ‘80%’, sperm and lower average motility, ‘40%’, sperm. See main text for further details.

Sperm were isolated from seminal plasma using DGC based on the methods outlined in Reynolds *et al.* (2017a) and summarised in Fig. 1. Briefly, this involved placing ~1 ml of liquefied semen on either 40% (v/v) (Process A for aims i and ii) or layered 40% and 80% (v/v) (Process B for aims iii) Percoll/PBS solution (Percoll, GE Healthcare Life Sciences, Little Chalfont, UK) in a 13 ml polypropylene tube with ventilation cap (Sarstedt, Leicester, UK). These were then centrifuged for 20 min at 300 *g* to produce an unfractionated pellet (Process A) or a population of sperm trapped at the 40–80% interface (termed ‘40%’ sperm) and those found at the bottom of the tube (termed ‘80%’ sperm) (Process B). In both cases, these sperm were re-suspended in PBS to at least three times their recovered volume before being centrifuged again for 10 min at 500 *g*. At each stage, the supernatant was removed, and the sperm was suspended in fresh PBS to a minimum volume of 600 μl.

### Baseline measurements

From each prepared sample, a 2.5 μl aliquot was placed in a 10 μm depth Leja chamber (Leja Products, Nieuw Vennep, the Netherlands) which was then placed on a heated plate at 37°C for 5 min before measuring concentration and motility using Sperm Class Analyzer, version 6 (Microptic SL, Barcelona, Spain) attached to a Microtec LM-2 Microscope (Mazurek Optical Services Ltd, Southam, UK) via a Basler acA1300-200uc camera (Basler AG, Ahrensburg, Germany). Since PBS does not contain any metabolites, sperm suspended in it generally swim poorly and so this process was repeated with a 20 μl aliquot of prepared sperm diluted 1 in 2 in PureSperm Wash (Nidacon, Gothenburg, Sweden) to assess the ability of the prepared sperm to swim when placed in a conventional medium. In addition, sperm vitality of each prepared sample was assessed using the LIVE/DEAD™ sperm viability kit (Fisher Scientific, Loughborough, UK), counting two replicates of at least 200 sperm as either alive (green) or dead (red) in order to establish the percentage of viable sperm.

### Sperm incubation with ^13^C substrates

In order to identify which ^13^C labelled substrates could be metabolised by sperm (aim i), 400 μl of unfractionated sperm (prepared by Process A in Fig. 1) was added to a 5 ml snap cap polystyrene round-bottom tube (Corning Falcon, Fisher Scientific) along with 15 μl antibiotics (10 000 units/ml penicillin and 10 mg/ml streptomycin diluted to 1/3 with PBS so that in tube concentrations were 90 units/ml penicillin and 90 μg/ml streptomycin, Sigma Aldrich) and 40 μl of 100 mM ^13^C labelled substrate (to give a final concentration of 8.8 mM). The substrates (obtained from either Sigma Aldrich or Cambridge Isotopes Laboratories, Tewksbury, MA, USA) tested were: ^13^C_u_-glucose, ^13^C_u_-fructose, ^13^C_1_-pyruvate, ^13^C_1_-butyrate, ^13^C_3_-lactate, ^13^C_2,4_-D-3-hydroxybutyrate, ^13^C_5_-l-glutamate (prepared from glutamic acid), ^13^C_1,2_-glycine and ^13^C_u_-galactose. Each substrate was incubated for 24 h at 37°C with eight samples of prepared sperm from individual men and where possible more than one substrate incubation was performed in parallel (in these cases the sperm were always shown to metabolise at least one substrate). After each incubation the sample was frozen at –80°C until MRS analysis (see below).

For each substrate found in aim (i) to be consistently metabolised by washed sperm, the rate constant was estimated for sperm from nine ejaculates to determine substrate kinetics (aim ii). Briefly, from each ejaculate, a 380 μl aliquot of unfractionated sperm (Fig. 1, Process A) was placed in a 5 mm MRS tube along with 40 μl of 100 mM ^13^C labelled substrate, 10 μl 200 mM ^13^C-urea (concentration and frequency reference), 20 μl D_2_O and 12 μl of antibiotics (as above). The tube was inserted into the MRS scanner which had been preheated to 37°C and a series of sequential ^13^C-spectra were then acquired approximately every 3 h (see below for details) until the change in the magnitude of the MRS peaks began to plateau, typically over a 18–48 h period.

To assess the effect of substrate concentration on sperm metabolism (aim ii), incubations were performed with unfractionated sperm (Fig. 1, Process A) and ^13^C labelled substrates consistently metabolised by sperm, identified in aim (i), (*n* = 3). From each ejaculate, 460 μl of unfractionated sperm, 15 μl of antibiotics and 80 μl of ^13^C labelled substrate diluted to a final concentration of 0, 1.8, 3.6, 7.2 or 14.4 mM was incubated for 4 h at 37°C in a 5 ml snap cap polystyrene round-bottom tube (Corning Falcon, Fisher Scientific). At the end of the incubation, each sample was frozen at −80°C until MRS analysis.

Metabolism differences between ‘40%’ and ‘80%’ sperm (aim iii), were examined by incubating 500 μl of each (prepared from individual samples using Process B shown in Fig. 1) with 15 μl of antibiotics and 40 μl of the 100 mM ^13^C labelled substrates, identified in aim (i) and confirmed in aim (ii), for 4 h at 37°C (*n* = 15). Samples were then frozen at −80°C until MRS analysis.

To assess the potential impact of any non-sperm cells present in the sperm fractions obtained for aim (iii), the concentration of non-sperm cells was determined according to the method outlined in WHO (2010). Briefly, 10 μl from each sperm preparation was smeared onto two polysine slides (Thermo Scientific, Saarbrücken Germany), and after air-drying stained with Diff-Kwik kit (Thermo Scientific) and imaged on a Microtec LM-2 microscope at 40× magnification. At least 400 sperm were counted along with any non-sperm cells observed in these fields of view; sperm heads without tails were excluded from the analysis. The concentration of non-sperm cells was determined using the formula in WHO (2010).

### Magnetic Resonance Spectroscopy (MRS)

All samples were scanned using a 9.4T Bruker Avance III MRS spectrometer (Bruker BioSpin GmbH, Karlsruhe, Germany), with a 5 mm broadband observe probe operating at either room temperature (21°C ± 0.5) for the frozen–thawed samples or at (37°C ± 0.5) for the experiments carried out for the rate constant experiments in aim (ii). Samples that were frozen at −80°C were thawed and 380 μl was placed in a 5 mm MRS tube (Norell, Morganton, NC, USA) with 20 μl D_2_O (Sigma Aldrich) and 10 μl of 200 mM ^13^C-urea (chemical shift and concentration reference, Sigma Aldrich) for MRS analysis. Spectra were acquired using a ^13^C{^1^H} inverse-gated pulse sequence (Spectral Width = 239 ppm, Number of acquisitions = 4096, Acquisition Time = 0.5 s, Delay Time = 2 s, Time domain points = 24036, flip angle = 16°). Each acquired spectrum was apodised with a 5 Hz exponential line broadening function, phase and baseline corrected using Bruker Topspin v2.1 software and referenced to the urea signal at a frequency offset δ = 165.5 ppm.

### Data analysis

For aim (i), all ^13^C-MRS spectra were first inspected visually by an expert in MRS (SR) for evidence of substrate metabolism, which could be identified by the appearance of new metabolite peaks and a visual reduction in the peak height of the ^13^C labelled substrate added. Identification of unknown peaks present in the spectra was assisted by reference to relevant metabolic pathways known to utilise the substrate and chemical shift values obtained from the human metabolome database version 4.0 (Wishart *et al.*, 2018).

All ^13^C-spectra peaks were integrated using the ‘trapz’ function in a custom Matlab script (R2017b, Mathworks, Natick, MA, USA) and predefined chemical shift integral ranges as appropriate for the substrate molecule (185.8–184.8 ppm, ^13^C_1_-lactate; 173.5–172.5 ppm, ^13^C_1_-pyruvate; 166.0–165.0 ppm, ^13^C-urea; 163.5–162.7 ppm, ^13^C-bicarbonate; 127.9–127.3 ppm, ^13^CO_2_; 99.2–98.2 ppm, ^13^C_u_-glucose; 102–99.6 ppm, ^13^C_u_-fructose; 71.5–70.5 ppm, ^13^C_2_-lactate; 23.4–22.4 ppm, ^13^C_3_-lactate). The integrals for peaks assigned to bicarbonate and carbon dioxide were summed to account for the biological equilibrium in which these molecules exist.

To examine rates of metabolism in aim (ii), each set of sequentially acquired ^13^C-spectra obtained from a single experiment were imported into Matlab and collated into sets of peak integral time courses obtained from each spectrum. Integrals versus time for each peak were plotted and fitted to either a mono-exponential growth (lactate and bicarbonate/CO_2_ peaks) or mono-exponential decay (glucose, fructose and pyruvate). Only fits to the data that had a Pearson correlation of *r* > 0.5 and a *P* < 0.01 were retained in order to avoid misestimation of rate values due to poor signal to noise (principally arising from the bicarbonate/CO_2_ integrals). The mean ± standard error (SE) was determined for each peak from each source substrate. Differences between metabolic rates were tested using a one-way ANOVA with Bonferroni post-hoc multi-comparison test *P* < 0.05 taken as significant.

The effect of concentration of supplied substrate on sperm metabolism (aim ii) was analysed by measuring lactate integrals for the integral from the ^13^C_1_ position (normalised by total sperm concentration) across the concentration ranges and comparing them using a Kruskal–Wallis test with *P* < 0.05 taken as significant.

The correlation between ^13^C_u_-glucose, ^13^C_u_-fructose or ^13^C_1_-pyruvate derived lactate integrals and total sperm concentration was determined by Pearson linear regression using GraphPad Prism (version 7.03, La Jolla, USA). The value of *r*^2^ and significance of the correlation are reported for the fit. A similar regression fit was also performed between lactate integral and non-sperm cell concentration.

In the comparison of metabolism by ‘40%’ and ‘80%’ sperm (aim iii), the spectra from co-incubation of ‘40%’ or ‘80%’ sperm with ^13^C substrates were initially phase- and baseline-corrected and referenced to the urea peak as above. Custom MatLab code was then used to integrate the lactate peak between 185.8 and 184.8 ppm and the bicarbonate peak between 163.5 and 162.7 ppm. These integrals were then normalised according to: (a) sperm concentration; (b) concentration of vital sperm; and (c) concentration of motile sperm (where the motility was determined for sperm in PureSperm wash at time zero – see above). In all of these normalisations, the concentration of sperm in PBS was used. Normalised substrate integrals were compared between ‘40%’ and ‘80%’ sperm by Mann–Whitney with *P* < 0.0055 taken as significant (0.05/9 as nine comparisons were done).

## Results

Semen samples from 97 men, recruited as part of a larger study, were used in these experiments; 52 were used in parallel for aims (i) and (ii) and 45 were used for aim (iii).

### Aim (i): Assessment of metabolically active substrates

**Figure 2:**
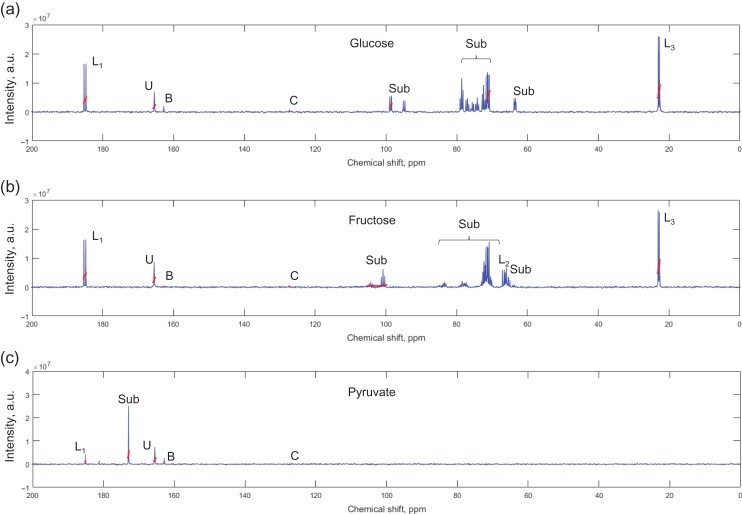
Example ^13^C MRS spectra for sperm incubated with: (**a**) ^13^C_u_-glucose; (**b**) ^13^C_u_-fructose; or (**c**) ^13^C_1_-pyruvate. Integral locations and widths are highlighted in red. Key: Sub – peaks from respective incubated substrate; L1, L2, L3 – lactate peaks, where the number indicates the carbon position, B – bicarbonate, C – carbon dioxide, U – urea.

The ability of ^13^C-MRS to detect human sperm metabolism was tested eight times, for each of the nine substrates associated with energy generation, over a 24 h incubation period. This showed that the metabolism of ^13^C substrates directly involved in the glycolytic pathway, glucose, fructose and pyruvate, were easily detected through conversion to lactate (Table I). Whilst conversion to lactate was always observed for these molecules, bicarbonate/CO_2_ was not (however, bicarbonate/CO_2_ peaks were observed in a subset of experiments from aim (ii) (Fig. 2) and aim (iii), see below). Incubation with ^13^C_3_-lactate showed shuttling to pyruvate in most samples (7 of 8) and this was often accompanied by production of a peak associated with an acetyl methyl group (6 of 8), which could be from dissociated acetyl-coA. Two of these eight experiments also showed ^13^C_3_-lactate metabolism to bicarbonate/CO_2_. Of the other ^13^C substrates, small quantities of acetoacetate were metabolised from ^13^C_2,4_-D-3-hydroxybutyrate, but further metabolism (including entry into the Krebs cycle) was not detected. There was no evidence of regular metabolism of ^13^C_1_-butyrate, ^13^C_5_-glutamate and ^13^C_1,2_-glycine by sperm, however, there was occasional production of bicarbonate by these substrates (Table I). Finally, in these eight samples, incubation with ^13^C_u_-galactose analysis by ^13^C-MRS showed only the original ^13^C_u_-galactose peaks. Example ^13^C-spectra from other 24 h incubations are shown in Supplementary Figs S1–S6.

**Table I:** Summary of observed metabolic products of human sperm after incubating with ^13^C labelled substrates.

	Number of spectra the metabolic product was observed in		
Incubated substrate	Lactate produced	Bicarbonate produced	Other metabolic products
^13^C_u_-glucose	8	0	–
^13^C_u_-fructose	8	0	–
^13^C_1_-pyruvate	8	0	–
^13^C_3_-lactate	N/A	2	Pyruvate (7 of 8), acetate (6 of 8)
^13^C_2,4_-D-3-hydroxybutyrate	0	2	Acetoacetate (8)
^13^C_1_-butyrate	0	1	Glutamate (2)
^13^C_5_-glutamate	0	2	–
^13^C_2_-glycine	0	1	–
^13^C_u_-galactose	0	0	–

Eight incubations were performed for each substrate at 37°C for 24 h with samples subsequently stored at −80°C prior to MRS analysis.

### Aim (ii): Substrate kinetics of human sperm

In order to establish the appropriate conditions for sperm/substrate incubations in MRS experiments ^13^C_u_-glucose, ^13^C_u_-fructose, ^13^C_1_-pyruvate and ^13^C_3_-lactate were selected for further analysis to examine their kinetics and optimum concentration for metabolism. Sperm from 31 ejaculates were used to determine rate constants: *n* = 9 each for ^13^C_u_-glucose, ^13^C_u_-fructose and ^13^C_1_-pyruvate; *n* = 4 for ^13^C_3_-lactate. Repeated sequential acquisition of spectra (*n* = 9 for ^13^C_u_-glucose, ^13^C_u_-fructose, ^13^C_1_-pyruvate; *n* = 2 for ^13^C_3_-lactate) showed mono-exponential growth and decay in product and source substrate respectively (see Supplementary Figs S7–S9). The rates of metabolite production and substrate consumption can be seen in Table II, excluding ^13^C_3_-lactate which did not provide reliable measures of metabolism.

**Table II:** Rate constants for ^13^C labelled substrate consumption by human sperm (*n* = 9 per substrate) and conversion to ^13^C-lactate and ^13^C-bicarbonate/CO_2_ were estimated from sequential ^13^C MRS spectra acquired at 37°C.

Rate measured per peak × 10^5^, s^−1^	Glucose (nf)	Fructose (nf)	Pyruvate (nf)
Incubated substrate	−1.2 ± 0.3 (8)	−0.9 ± 0.2 (9)	−10.8 ± 6.3 (7)
^13^C_1_-lactate	1.7 ± 0.4 (9)	1.4 ± 0.2 (9)	5.0 ± 0.8 (8)
Bicarbonate	2.4 ± 0.2 (2)	N/A	5.9 ± 0.9 (7)

Peak integrals were fitted to a mono-exponential, where a negative rate constant indicates consumption of substrate. Mean ± SE values shown where only fits with a Pearson correlation, *r* > 0.5 and significance *P* < 0.01 were retained (number of retained fits shown in parenthesis). See method for details.

nf: number of retained fits of 9.

Universally isotopically labelled ^13^C_u_-glucose and ^13^C_u_-fructose will label all three carbons of lactate whereas ^13^C_1_-pyruvate will only be converted to ^13^C_1_-lactate. An ANOVA test with Bonferroni post-hoc test showed that the choice of lactate peak (1, 2 or 3) had no significant effect on the estimated rate constant derived from ^13^C_u_-glucose (*P* = 0.99) or ^13^C_u_-fructose (*P* = 0.69). Given that a fructose MRS peak obscures the C2 labelled position of lactate and the pyruvate labels only C1, therefore, only the ^13^C_1_ peak of lactate was used for subsequent analysis. This showed that there was no significant difference (ANOVA with Bonferroni post-hoc test) in the rate of ^13^C_u_-glucose (1.7 ± 0.4 × 10^−5^ s^−1^) or ^13^C_u_-fructose (1.4 ± 0.2 × 10^−5^ s^−1^) conversion to lactate. However, the single enzymatic step of ^13^C_1_-pyruvate to lactate (5.0 ± 0.8 × 10^−5^ s^−1^) was approximately three times faster than that of ^13^C_u_-glucose and ^13^C_u_-fructose (*P* = 0.0011 and *P* = 0.00042, respectively). Visually inspecting the rate data showed that typically ^13^C_1_-pyruvate incubations reached over half the maximum lactate production by the second time point (an average of 4.5 h into the experiment), whereas from ^13^C_u_-glucose and ^13^C_u_-fructose, over half the maximum lactate production was often reached at the sixth time point (an average of 19.5 h into the incubation).

In contrast to 24 h incubations (aim i), bicarbonate was produced during some rate experiments (aim ii). It appeared more frequently from ^13^C_1_-pyruvate (6 of 8) than ^13^C_u_-glucose (3 of 8), although the rate constants for these were not significantly different. This was probably due to the smaller peak intensity causing large standard error in the rate constant (see Table II). ^13^C_u_-fructose did not produce bicarbonate under these conditions. Sequential spectra were also acquired where ^13^C_3_-lactate was the source substrate, however, in these experiments no consistent build-up of pyruvate or bicarbonate/CO_2_ was observed (*n* = 4), most likely due to the low signal to noise of the pyruvate peak that had been observed for ^13^C_3_-lacate from aim (i). A long incubation allows time for MRS peaks to increase leading to a reduction in integration errors due to signal noise. However, sperm quality also degrades with time, therefore, 4 h was chosen for subsequent experiments.

**Figure 3:**
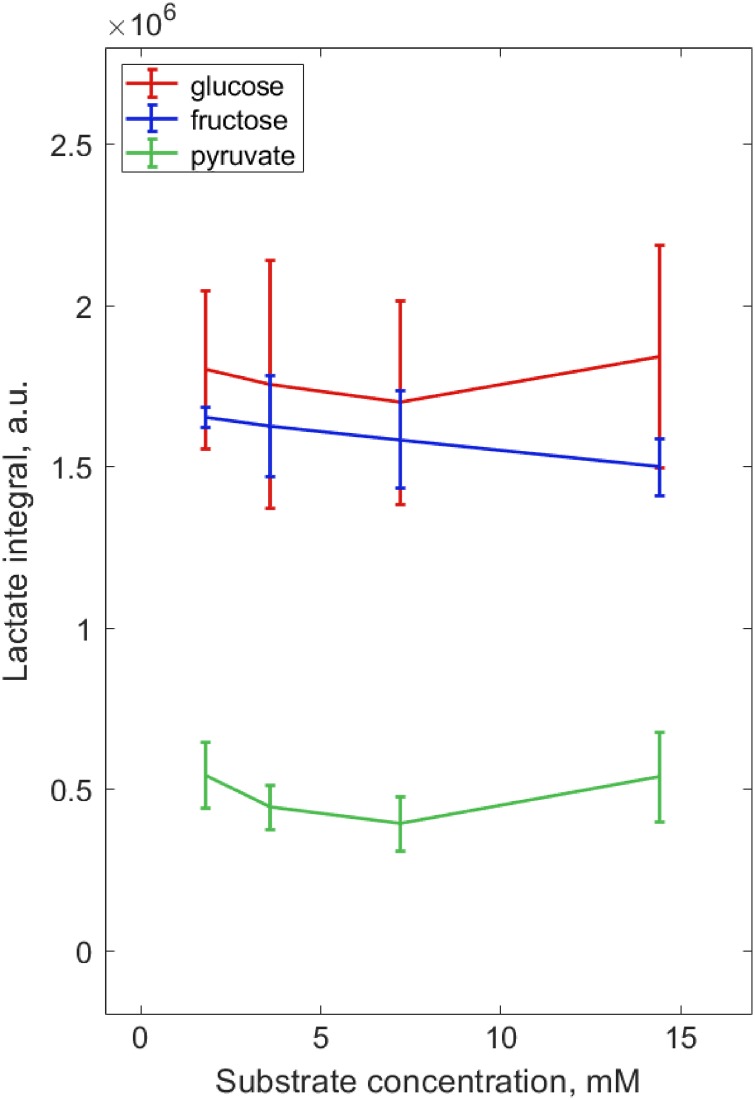
Normalising the lactate integral for sperm concentration and plotting against substrate concentration showed that sperm metabolism was not limited by substrate availability as tested by Kruskal–Wallis. Sperm were tested over a 4 h incubation (*n* = 3) with ^13^C_u_-glucose, ^13^C_u_-fructose or ^13^C_1_-pyruvate (shown in blue, red and green respectively) at 1.8 mM, 3.6 mM, 7.2 mM and 14.4 mM concentrations.

The optimum metabolite concentrations were assessed during a 4 h incubation for the principal metabolic substrates of ^13^C_u_-glucose, ^13^C_u_-fructose and ^13^C_1_-pyruvate (three washed sperm samples per substrate). Overall, the amount of lactate produced by sperm (normalised to total sperm concentration) was not significantly influenced by the supplied substrate concentration (1.8–14.4 mM) (see Fig. 3 and Supplementary Fig. S10). However, after 4 h of incubation the remaining substrate peaks for the lowest concentration, 1.8 mM, were almost absent from the MRS spectrum. Consequently, to ensure substrate metabolism was not limited by its availability and to allow for higher sperm concentration than used here in subsequent experiments, a concentration of 7.2 mM was selected for the experiments in aim (iii).

### Aim (iii): Substrate metabolism by ‘40%’ and ‘80%’ sperm

Sperm metabolism was assessed using sperm from 45 individual ejaculates that had been separated by DGC (Process B in Fig. 1). This method yields two sperm populations (termed ‘40%’ and ‘80%’ sperm in Reynolds *et al.*, 2017a) with significant differences in both motility and vitality (see Table III). The percentage sperm motility were not similar to the viability (i.e. sperm motility was not entirely due to a lack of viable sperm) and, importantly for our study, there was no significant difference in sperm concentration between the ‘40%’ and ‘80%’ groups.

**Table III:** Initial characteristics of sperm separated by DGC into two pellets ‘40%’ and ‘80%’ sperm (mean ± standard deviation, except non-sperm cells which show median and range) measured.

	80% sperm (*n* = 45)	40% sperm (*n* = 45)	Difference
Concentration, 10^6^/ml			
Sperm	43.8 ± 23.4	42.1 ± 25.0	1.7
NS cell	0.4**** (0.0–4.5)	1.1**** (0.1–6.9)	0.7
Total vitality,%			
	65.0 ± 13.8***	55.0 ± 10.0***	10.0
Total motility, %			
	42.9 ± 20.9***	29.5 ± 13.8***	13.4

A two independent samples *t*-test with Welch’s correction was used for data that passed a Gaussian distribution (concentration, motility and vitality) else Mann–Whitney test (non-sperm cell concentration) was used to determine differences between ‘40%’ and ‘80%’ sperm populations, *** *P* < 0.001, **** *P* < 0.0001.

The ‘40%’ and ‘80%’ sperm samples also contained small numbers of other cell types (e.g. leucocyte, germ and epithelial cells) that may have been metabolically active. Therefore, it was important to determine their prevalence in the sperm preparations assessed in aim (iii). Briefly, there were more non-sperm cells in the ‘40%’ compared to the ‘80%’ sperm population (median: 1.1, range: 0.1–6.9 vs. median: 0.4, range: 0.0–4.5 × 10^6^/ml, *P* < 0.0001, respectively). Both fractions showed a correlation between the concentration of non-sperm cells and sperm concentration but the *r*^2^ was higher for the ‘40%’ sperm fraction (*P* = 0.0018, *r*^2^ = 0.21), than the ‘80%’ fraction (*P* = 0.046, *r*^2^ = 0.09), see supplementary Fig. S11.

The ‘40%’ and ‘80%’ sperm were separated into cohorts incubated with different substrates (*n* = 15 samples per sperm population) with sperm concentration, vitality and motility measured at the start of incubation (Table IV). A Kruskal–Wallis test with a Dunn’s multiple comparison correction showed that none of these parameters were significantly different for the ‘80%’ or the ‘40%’ sperm for any substrate.

**Table IV:** Characteristics of ‘40%’ and ‘80%’ sperm populations, separated by incubated ^13^C substrate (mean ± standard deviation).

	^13^C_u_-glucose		^13^C_u_-fructose		^13^C_1_-pyruvate	
	80% sperm (*n* = 15)	40% sperm (*n* = 15)	80% sperm (*n* = 15)	40% sperm (*n* = 15)	80% sperm (*n* = 15)	40% sperm (*n* = 15)
Concentration, 10^6^/ml						
Sperm	48.7 ± 25.8	50.3 ± 28.3	43.4 ± 28.0	34.2 ± 20.5	39.1 ± 15.1	41.6 ± 24.6
Total vitality,%						
0 h	63.6 ± 15.1	52.8 ± 8.4	66.0 ± 13.7	59.3 ± 9.1	65.4 ± 13.2	52.9 ± 11.5
Total motility, %						
0 h	40.9 ± 23.1	28.0 ± 12.7	42.3 ± 21.2	33.4 ± 14.4	45.5 ± 19.5	27.3 ± 14.2

Concentration, vitality and motility were measured the start of incubations. Differences between ‘40%’ and ‘80%’ sperm populations were tested using a Kruskal–Wallis with Dunn’s multiple comparison test. No significant differences were found.

**Figure 4:**
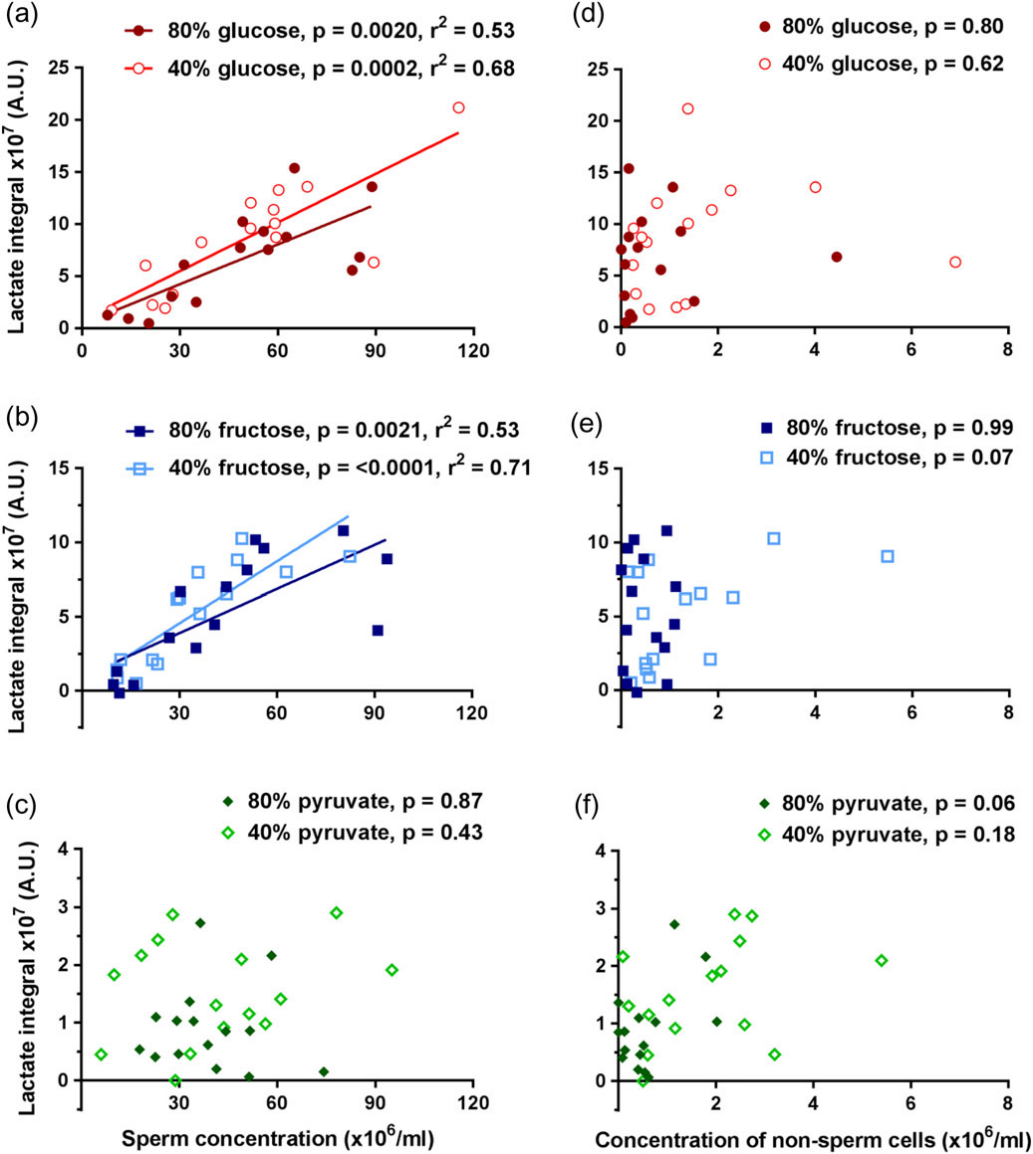
Comparing lactate integral correlation with sperm and non-sperm cell concentrations. Sperm concentrations (*n* = 15) correlated with lactate integrals for (**a**) ^13^C_u_-glucose, (**b**) ^13^C_u_-fructose and (**c**) ^13^C_1_-pyruvate incubations. Non-sperm cell concentrations did not correlate with lactate integrals for (**d**) ^13^C_u_-glucose and (**e**) ^13^C_u_-fructose or (**f**) ^13^C_1_-pyruvate incubations. Lines of best fit are shown for correlations where *P* < 0.05 for slope being non-zero.

Lactate peaks in the ^13^C-MRS spectra were plotted against total sperm concentration to determine whether the observed lactate arose from sperm metabolism. When sperm were incubated with either ^13^C_u_-glucose or ^13^C_u_-fructose, there was a significant strong linear correlation with total sperm concentration (see Fig. 4a and b). However, the lactate integrals obtained from sperm incubated with ^13^C_1_-pyruvate showed no correlation (*P* > 0.05) with concentration for either ‘40%’ or ‘80%’ sperm populations (Fig. 4c). Importantly, no correlations were found when lactate peak integrals were plotted against non-sperm cell concentrations (Fig. 4d, e, f), strongly suggesting that non-sperm cells did not make a significant contribution to the metabolism observed (*P* > 0.05).

**Figure 5:**
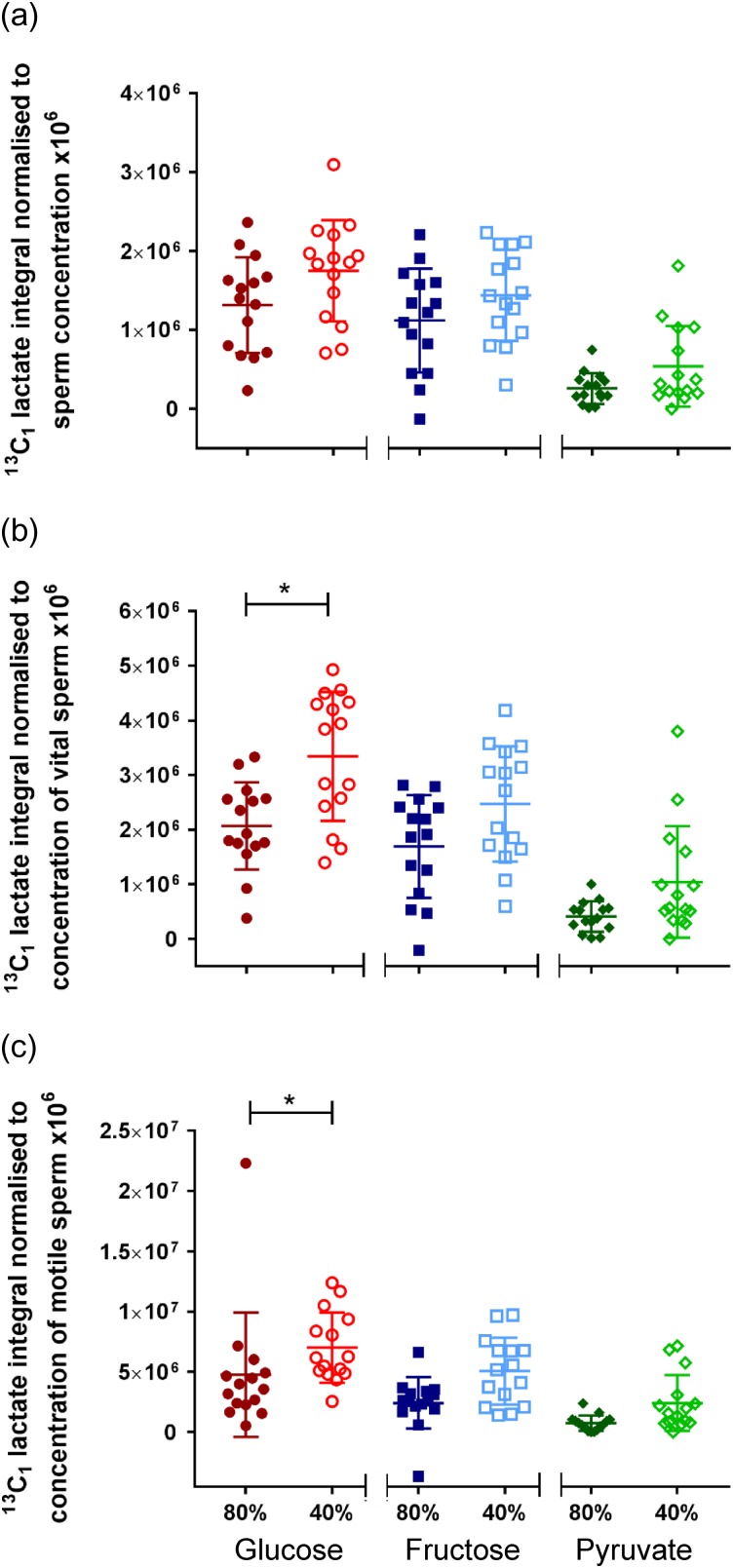
Comparing ^13^C_u_-glucose, ^13^C_u_-fructose or ^13^C_1_-pyruvate metabolism (*n* = 15 per substrate) between ‘40%’ and ‘80%’ sperm. Integrals were measured for ^13^C_1_-lactate and normalised against either (**a**) sperm concentration, (**b**) concentration of vital sperm or (**c**) concentration of motile sperm.

After a 4 h incubation at 37°C, lactate peaks could be identified from all substrates metabolised by ‘40%’ and ‘80%’ sperm (*n* = 15). The peak integral measured from its MRS spectrum represents an absolute concentration of the metabolite present in solution, which is a function of the sperm concentration as seen in Fig. 4. We reasoned vital and motile sperm would have larger impacts on metabolism than immotile or dead sperm and therefore normalised lactate integrals by three different methods: total sperm concentration (Fig. 5a), concentration of vital sperm (Fig. 5b) and concentration of motile sperm (Fig. 5c). Briefly, the lactate integral was only significantly higher for ^13^C_u_-glucose incubations with ‘40%’ compared to ‘80%’ sperm when normalised to vital concentration (3.3 ± 0.3 × 10^6^ vs 2.0 ± 0.21 × 10^6^; *P* = 0.0049) or motile concentration (7.0 ± 0.75 × 10^6^ vs 4.8 ± 1.3 × 10^6^; *P* = 0.0032), Mann–Whitney test (*P* < 0.0055 taken as statistically significant). No significant differences were found for these sperm populations incubated with either ^13^C_u_-fructose or ^13^C_1_-pyruvate or in any incubation when normalised to total sperm concentration.

After a 4 h incubation at 37°C, bicarbonate, a marker of oxidative phosphorylation, was occasionally observed. Bicarbonate was produced from ^13^C_1_-pyruvate metabolised by ‘40%’ sperm (5 occurrences) and ‘80%’ sperm (3 occurrences), ^13^C_u_-glucose metabolised by ‘40%’ sperm (2 occurrences) and ‘80%’ sperm (2 occurrences) and ^13^C_u_-fructose metabolised by ‘40%’ sperm (2 occurrences). The appearance of bicarbonate was associated across a range of sperm concentrations (mean 59.2 × 10^6^/ml, range 17.9–115.4 × 10^6^/ml) and also typical non-sperm cell concentrations (mean 1.2 × 10^6^/ml range 0–2.8 × 10^6^/ml) so bicarbonate appearance was not associated with high sperm or non-sperm cell concentrations. The sporadic nature of bicarbonate production and low signal to noise for this peak meant that no significant differences were observed for bicarbonate integrals from ‘40%’ and ‘80%’ sperm.

## Discussion

This paper continues our previous work that used ^1^H-MRS to investigate the endogenous metabolites present in human sperm (Reynolds *et al.*, 2017a). Here we examine human sperm metabolism of a range of exogenous ^13^C labelled substrates by ^13^C-MRS and define the experimental conditions necessary to observe them. The chosen metabolites feed into glycolysis and oxidative phosphorylation at various locations in the metabolic pathways and they included sugars, ketone bodies, fatty acids and amino acids. Glucose, fructose, pyruvate and lactate were chosen for their role in glycolysis and their predominance in the female reproductive tract and seminal fluid (Weed and Carrera, 1970; Ford and Rees, 1990; Andrade-Rocha, 1999), with galactose also included as its enzyme, galactose-1-phosphate uridylyltransferase, has been predicted to be important for sperm motility (Asghari *et al.*, 2017). Ketone body D-3-hydroxybutyrate was selected due to its ability to maintain and restore motility of mouse sperm (Tanaka *et al.*, 2004) and the fatty acid analogue butyrate was chosen as it had previously been demonstrated to be metabolised by ram, bull, dog and fowl spermatozoa (Scott *et al.*, 1962). Finally, amino acids glycine and glutamate feed into different aspects of oxidative phosphorylation and are common amino acids found in seminal fluid which may have a role in protecting sperm motility in bovine and ram spermatozoa (Tyler and Tanabe, 1952; Setchell *et al.*, 1967; Kondo, 1975).

In these experiments, we found that ^13^C_u_-glucose and ^13^C_u_-fructose were predominately metabolised to lactate which is indicative of glycolysis in a process that also produces two net units of ATP per carbohydrate molecule. The end point of glycolysis (i.e. ^13^C_1_-pyruvate) was also mainly converted to lactate. The enzyme lactate dehydrogenase catalyzes a shuttling reaction between pyruvate and lactate to maintain cofactors NADH/NAD^+^ to support further glycolysis and the electron transport chain as required for cellular function.

The relative magnitude of the lactate and pyruvate ^13^C-MRS peaks demonstrate an important feature of these experiments. The degree of ^13^C-lactate or ^13^C-pyruvate observed by ^13^C-MRS is dependent not only on enzyme activity and substrate preference but also the endogenous concentration of unlabelled lactate/pyruvate. This is because any ^13^C-lactate generated from ^13^C-pyruvate can, in principle, be converted back to ^13^C-pyruvate. However, where there is a large concentration of unlabelled ^12^C-lactate present then there is a great probability of those molecules being converted back to pyruvate, leading to ^13^C-lactate being retained within the lactate pool. Conversely, for ^13^C_3_-lactate sperm incubations, the lower endogenous ^12^C-pyruvate concentration resulted in fewer ^13^C-labelled pyruvate molecules being retained leading to smaller MRS pyruvate signal. Hence, how metabolites are enzymatically exchanged and their endogenous concentration affects the ^13^C MRS observability of intermediate metabolites within a metabolic pathway.

In addition to pyruvate, ^13^C_3_-lactate often produced a small acetate peak. In mammalian cells, pyruvate can be metabolised to acetyl-CoA by pyruvate dehydrogenase and then to acetate by acetyl-CoA hydrolase but this will not generate ATP (Knowles *et al.*, 1974). Unlike for ^13^C_u_-glucose and ^13^C_u_-fructose, ^13^C-MRS was not able to detect human sperm metabolism of ^13^C_u_-galactose. ^14^C galactose studies have reported that this molecule is not metabolised by human sperm (MacLeod, 1941; Rogers and Perreault, 1990), however, it has been reported to undergo slow glycolysis in human semen (Mann, 1946). It is likely that if galactose is metabolised by human sperm then its concentration falls below the sensitivity of conventional MRS.

^13^C_2,4_-D-3-hydroxybutyrate was converted to acetoacetate catalysed by β-hydroxybutyrate dehydrogenase, an enzyme that in other tissues (Smith *et al.*, 1969), including rat testis (Niemi and Ikonen, 1962), can be switched on by environmental cues, such as starvation. The reversible conversion of hydroxybutyrate to acetoacetate is linked to NADH generation and calcium uptake in bovine epididymal sperm (Vijayaraghavan *et al.*, 1989) and to supporting motility in mouse sperm (Tanaka *et al.*, 2004). Although acetoacetate can feed into the Krebs cycle, no evidence for this was observed. Finally, there were two instances of ^13^C_1_-butyrate conversion to glutamate. It is possible that glutamate production from ^13^C_1_-butyrate was through Krebs cycle metabolism; however, as there were no anaplerotic markers in the MRS spectrum, the exact mechanism for generation of glutamate remains unknown.

Bicarbonate was occasionally produced from incubations with ^13^C_1_-butyrate, ^13^C_3_-lactate, ^13^C_2,4_-D-3-hydroxybutyrate, ^13^C_5_-l-glutamate or ^13^C_1,2_-glycine after 24 h, but under the same conditions ^13^C_u_-glucose, ^13^C_u_-fructose or ^13^C_1_-pyruvate did not show bicarbonate production. In aims (ii) and (iii), bicarbonate and carbon dioxide production was most often observed from ^13^C_1_-pyruvate and, to a lesser extent, ^13^C_u_-glucose, and least often from ^13^C_u_-fructose. Generally, the production of carbon dioxide/bicarbonate by sperm samples was intermittent and why some samples produced measurable ^13^C-bicarbonate and others did not remains unclear as there was no obvious visible bacteria, fungi, high sperm concentration or a high proportion of non-sperm cells in these samples. Bicarbonate plays an important role in sperm capacitation (Miraglia *et al.*, 2010) and its role in relationship to these substrates and capacitation warrants further investigation.

These experiments were conducted under atmospheric oxygen and carbon dioxide levels which is likely to have influenced which metabolic pathways were selected. Atmospheric oxygen and carbon dioxide levels were chosen for practical reasons while using the 9.4T scanner. These conditions would provide more oxygen and less carbon dioxide than would be expected *in vivo* (Ng *et al.* 2018), which should promote the aerobic use of OxPhos. Regardless of these conditions, OxPhos was still only recorded at low levels, which does seem to suggest that human sperm inherently prefer anaerobic glycolysis to meet their energy needs. This is supported by Hereng *et al.* (2011) who also did not observe OxPhos metabolism in human sperm and commented that they used glycolysis.

Rate constants for ^13^C_u_-glucose and ^13^C_u_-fructose conversion to lactate were similar, suggesting that transport across the cellular membrane was not a limiting factor. The estimated rate constant for ^13^C_1_-pyruvate to lactate was much faster than ^13^C_u_-glucose/^13^C_u_-fructose conversion to lactate. This is to be expected as the rate limiting step for glycolysis is catalysed by phosphofructokinase which will affect metabolism of both ^13^C_u_-glucose and ^13^C_u_-fructose, but not ^13^C_1_-pyruvate. No significant differences were measured for bicarbonate rate constants estimated for ^13^C_u_-glucose, ^13^C_u_-fructose or ^13^C_1_-pyruvate, due to large errors probably resulting from the low signal to noise observed for this molecule. It was not possible to obtain rates measurements for ^13^C_3_-lactate incubations, due to low concentrations of pyruvate being produced. Whilst incubating sperm with ^13^C labelled substrate for longer would increase the magnitude of the peaks in MRS spectrum, there was a concern that, over extended times, sperm death would confound the results. An incubation time of 4 h was chosen for subsequent experiments where the build-up of metabolic product from the rates constant experiments in aim (ii) was approximately linear.

The concentration of ^13^C labelled substrates chosen for further experiments was 7.2 mM for each incubation. This is within a similar concentration range to that used by Williams and Ford (2001) who found that the optimal *in-vitro* concentration of glucose was 5.56 mM for supporting human sperm motility. ^13^Cu-glucose, ^13^C_u_-fructose and ^13^C_3_-lactate were within physiological levels experienced by sperm either within seminal plasma or the female reproductive tract (Ford and Rees, 1990) (Gardner *et al.*, 1996). Pyruvate is found at slightly lower concentrations in seminal plasma (1–6 mM) (Mann and Lutwak-Mann, 1981) and in the female reproductive (0.1–0.2 mM) (Gardner *et al.*, 1996; Tay *et al.*, 1997). Therefore, our examinations were within optimal concentration ranges to support motility for some substrates, but were super-physiological for pyruvate. Our experiments used each substrate in isolation and interaction between sperm and multiple substrates is likely to be different (Hereng *et al.*, 2011) both *in vitro* and in the female reproductive tract.

During preparation for assisted conception, the ‘40%’ sperm are normally discarded as they tend to be of poorer quality and also are co-localised with a higher proportion of non-sperm cells (Henkel and Schill, 2003). However, these sperm are still biologically relevant, as *in vivo* all sperm are deposited in the female reproductive tract and ‘40%’ sperm may represent a substantial fraction of the sperm population in men with male factor infertility. As in our previous work using ^1^H-MRS (Reynolds *et al.*, 2017a), we exploited the difference between these two sub-populations to test the ability of ^13^C-MRS to detect metabolism differences. First it was important to consider the metabolic role of any non-sperm cells but, unlike that for sperm, the concentration of non-sperm cells did not correlate with the lactate integral, suggesting that they have a minimal effect on recorded lactate production at the concentrations we observed for them. Therefore, the highest non-sperm cell concentration in this analysis, of 6.9 × 10^6^/ml, was taken as the limit for non-sperm cell concentrations known not to affect sample metabolism measurably. Metabolism of ^13^C-labelled substrates by the differing non-sperm cells types found in seminal plasma could be done in future studies.

Both ‘40%’ and ‘80%’ sperm produced similar amounts of ^13^C-lactate when normalised to total sperm concentration, however, unlike ^1^H-MRS where sperm concentration will affect metabolite detection regardless of vitality, ^13^C-MRS will only detect the ^13^C-products of metabolising sperm. Therefore, normalising the lactate signal by the vital sperm concentration was considered more appropriate. Additionally, as sperm motility is estimated to account for 70% of the sperm’s total ATP production (Rikmenspoel, 1965), normalisation of the lactate signal by motile sperm concentration was also performed. For either of these normalisations, it was found that ‘40%’ sperm incubated with ^13^C_u_-glucose produced more lactate, i.e. a larger metabolic output, than the equivalent ‘80%’ sperm, whereas fructose or pyruvate incubations showed no significant differences between ‘40%’ and ‘80%’ sperm.

It is interesting that only measurements after incubation with ^13^C_u_-glucose showed a difference between motile or vital ‘40%’ and ‘80%’ sperm. Kinetic experiments presented here suggest that ^13^C_u_-fructose and ^13^C_u_-glucose were metabolised by glycolysis to similar levels and should have similar effects on sperm function. Sperm are highly polarised cells and one reason for a difference between the ability of glucose and fructose to support sperm motility could be related to the roles of differing hexose transporter and their distribution within sperm (Angulo *et al.*, 1998; Bucci *et al.*, 2011). In turn these molecules may support different phases of sperm life (du Plessis *et al.*, 2015).

There are many reasons why ‘80%’ sperm might show lower lactate production than ‘40%’ sperm. The ‘40%’ sperm are more likely to have abnormal morphology with increased cytoplasm (Aitken and West, 1990). As glycolysis is an uncontrolled reaction that takes place in the cytoplasm, simply having more cytoplasm may produce more lactate. Conversely, tightly controlled energy production in ‘80%’ sperm may limit ROS production and subsequent DNA damage (Aitken *et al.*, 1996).

In conclusion, we have examined sperm metabolism by ^13^C-MRS and found metabolic differences in sub-populations from the same individuals (‘80%’ vs. ‘40%’ sperm). Human sperm, from either sub-population, seem to predominantly use the glycolytic pathway to meet their energy needs when supplied with ^13^C_u_-glucose or ^13^C_u_-fructose. The level of glycolysis was increased for ‘40%’ sperm incubated with ^13^C_u_-glucose compared to equivalent ‘80%’ sperm, which perhaps suggests that poor quality sperm are metabolically noisy. By implication, this may also be true of poor quality sperm from sub-fertile men and further work to examine this using MRS technology may help to better unravel the metabolic characteristics of poor quality sperm.

## Supplementary Material

Supplementary Data

Supplementary Data

Supplementary Data

Supplementary Data

Supplementary Data

Supplementary Data

Supplementary Data

Supplementary Data

Supplementary Data

Supplementary Data

Supplementary Data

Supplementary Data
