# Supplementary material for: Probing human sperm metabolism using 13C-magnetic resonance spectroscopy
Source: Mol Hum Reprod. 2018 Nov 3;25(1):30–41. doi: 10.1093/molehr/gay046 (PMC6314230; doi:10.1093/molehr/gay046)
Supplement: Supplementary Data [file gay046_legends_to_supplementary_figures.docx]

**Legends to supplementary figures:**

Supplementary Figure S1: Example ^13^C-MRS spectrum of ^13^C_3_-lactate after a 24h incubation with sperm at 37^o^C. Peak assignment: S - 22.8 ppm is ^13^C_3_-lactate (source substrate); P - 29.2 ppm is ^13^C_3_-pyruvate; A - 26.0 ppm is ^13^C_2_-acetate; U - 165.5 ppm is ^13^C-urea (added reference compound post incubation).

Supplementary Figure S2: Example ^13^C-MRS spectrum of ^13^C_2,4_-D-3-hydroxybutyrate after a 24h incubation with sperm at 37^o^C. Peak assignment: S_2_ - 49.2 ppm & S_4_ - 24.5 ppm is ^13^C_2,4_-D-3-hydroxybutyrate (source substrate); B_2_ - 55.9 ppm & B_4_ - 32.3 ppm is ^13^C_2,4_-acetoacetate; U - 165.5 ppm is ^13^C-urea (added reference compound post incubation).

Supplementary Figure S3: Example ^13^C-MRS spectrum of ^13^C_1_-butyrate after a 24h incubation with sperm at 37^o^C. Peak assignment: S - 186.8 ppm is ^13^C_1_-butyrate (source substrate); G - 184.1 ppm is ^13^C_5_-glutamate; U - 165.5 ppm is ^13^C-urea (added reference compound post incubation).

Supplementary Figure S4: Example ^13^C-MRS spectrum of ^13^C_1,2_-glycine after a 24h incubation with sperm at 37^o^C. Peak assignment: S_1_ - 175.1 ppm & S_2_ - 44.1 ppm is ^13^C_1,2_-glycine (source substrate); U - 165.5 ppm is ^13^C-urea (added reference compound post incubation).

Supplementary Figure S5: Example ^13^C-MRS spectrum of ^13^C_5_-glutamate after a 24h incubation with sperm at 37^o^C. Peak assignment: S - 184.0 ppm is ^13^C_5_-glutamate (source substrate); U -165.5 ppm is ^13^C-urea (added reference compound post incubation).

Supplementary Figure S6: Example ^13^C-MRS spectrum of ^13^C_u_-galactose after a 24h incubation with sperm at 37^o^C. Peak assignment: S between 104 - 62 ppm is ^13^C_u_-galactose (source substrate); U - 165.5 ppm is ^13^C-urea (added reference compound post incubation).

Supplementary Figure S7: (a) Example ^13^C-MRS spectrum of ^13^C_u_-fructose after at least 24h incubation with sperm at 37^o^C in the 9.4 T MRS spectrometer. Peak assignment: S is ^13^C_u_-fructose (source substrate between 104 - 62 ppm); L_1_ - 185.3 ppm, L_2_ - 71.2 ppm and L_3_ – 22.8 ppm is for ^13^C-lactate labelled in the 1, 2 or 3 position respectively; B – 162.0 ppm is bicarbonate; C – 128.0 ppm is carbon dioxide; U - 165.5 ppm is ^13^C-urea (added reference compound). Peak integral location and range are shown as red markers. (b) Repeated spectra, acquired every 3 hours, are shown transitioning from blue to red (linearly right shifted to aid visualisation with time axis shown above a peak). Live sperm metabolism can be seen in these spectra as lactate peaks increase in size and fructose peaks decrease. (c) Integrals from ^13^C_1_-lactate, bicarbonate and carbon dioxide (summed over both integrals), ^13^C_u_-fructose and ^13^C_3_-lactate were repeatedly measured over time and fitted to an exponential growth curve or decay curve, as appropriate. These show how rate values were calculated from ^13^C_u_-fructose incubations for lactate increase and ^13^C_u_-fructose decline.

Supplementary Figure S8: (a) Example ^13^C-MRS spectrum of ^13^C_u_-glucose after at least 24h incubation with sperm at 37^o^C in the 9.4 T MRS spectrometer. Peak assignment: S between 104 - 67.0 ppm & at 65 ppm is ^13^C_u_-glucose (source substrate); L_1_ - 185.3 ppm, L_2_ - 71.2 ppm and L_3_ – 22.8 ppm is ^13^C-lactate; B – 162.0 ppm is bicarbonate; C – 128.0 ppm is carbon dioxide; U - 165.5 ppm is ^13^C-urea (added reference compound). Peak integral location and range are shown as red markers. (b) Repeated spectra, acquired every 3 hours, are shown transitioning from blue to red (linearly right shifted to aid visualisation with time axis shown above a peak). Live sperm metabolism can be seen in these spectra as lactate peaks increase in size and glucose peaks decrease. (c) Integrals from ^13^C_1_-lactate, bicarbonate and carbon dioxide (summed over both integrals), ^13^C_u_-glucose ^13^C_2_-lactate and ^13^C_3_-lactate were repeatedly measured over time and fitted to an exponential growth curve or decay curve, as appropriate. These show how rate values were calculated from ^13^C_u_-glucose incubations for lactate and carbon dioxide increase and ^13^C_u_-glucose decline.

Supplementary Figure S9: (a) Example ^13^C-MRS spectrum of ^13^C_u_-pyruvate after at least 24h incubation with sperm at 37^o^C in the 9.4 T MRS spectrometer. Peak assignment: S 172.9 ppm is ^13^C_1_-pyruvate (source substrate); L_1_ - 185.3 ppm is ^13^C-lactate; B – 162.0 ppm is bicarbonate; C – 128.0 ppm is carbon dioxide; U - 165.5 ppm is ^13^C-urea (added reference compound) (a). Peak integral location and range are shown as red markers. (b) Repeated spectra, acquired every 3 hours, are shown transitioning from blue to red (linearly right shifted to aid visualisation with time axis shown above a peak). Live sperm metabolism can be seen in these spectra as lactate peaks increase in size and pyruvate peaks decrease. (c) Integrals from ^13^C_1_-lactate, bicarbonate and carbon dioxide (summed over both integrals), ^13^C_1_-pyruvate were repeatedly measured over time and fitted to an exponential growth curve or decay curve, if appropriate. These show how rate values were calculated from ^13^C_1_-pyruvate incubations for lactate and carbon dioxide increase and ^13^C_1_-pyruvate decline.

Supplementary Figure S10: Example spectra of substrates incubated with sperm at different concentrations for 4 hours. (a) ^13^C_u_-glucose, (b) ^13^C_u_-fructose and (c) ^13^C_1_-pyruvate spectra are shown with higher concentrations of ^13^C labelled substrate (linearly right shifted to aide visualisation, 0 mM – black spectra, 1.8 mM – orange spectra, 3.6 mM – blue spectra, 7.2 mM – green spectra and 14.4 mM – yellow spectra). The 0 mM spectra showed no substrate present or lactate created. All other concentrations produced lactate (L_1_ & L_3_) at similar level. However, levels of added substrate (S) depended upon the amount of substrate added and were sometimes harder to observe at the lower concentrations. ; U - 165.5 ppm is ^13^C-urea (added reference compound). ; H – 181.1 ppm is ^13^C_1_-hydroxypyruvate (non-metabolically active compound in aqueous equilibrium with pyruvate)

Supplementary Figure S11: Plotting the concentration of non-sperm cells against sperm concentration. Lines of best fit and r values are shown for correlations with p < 0.05 for slope being non-zero, grey for ‘40%’ sperm values and black for ‘80%’ sperm.
