# Supplementary figures and images for: Probing human sperm metabolism using 13C-magnetic resonance spectroscopy
Source: Mol Hum Reprod. 2018 Nov 3;25(1):30–41. doi: 10.1093/molehr/gay046 (PMC6314230; doi:10.1093/molehr/gay046)

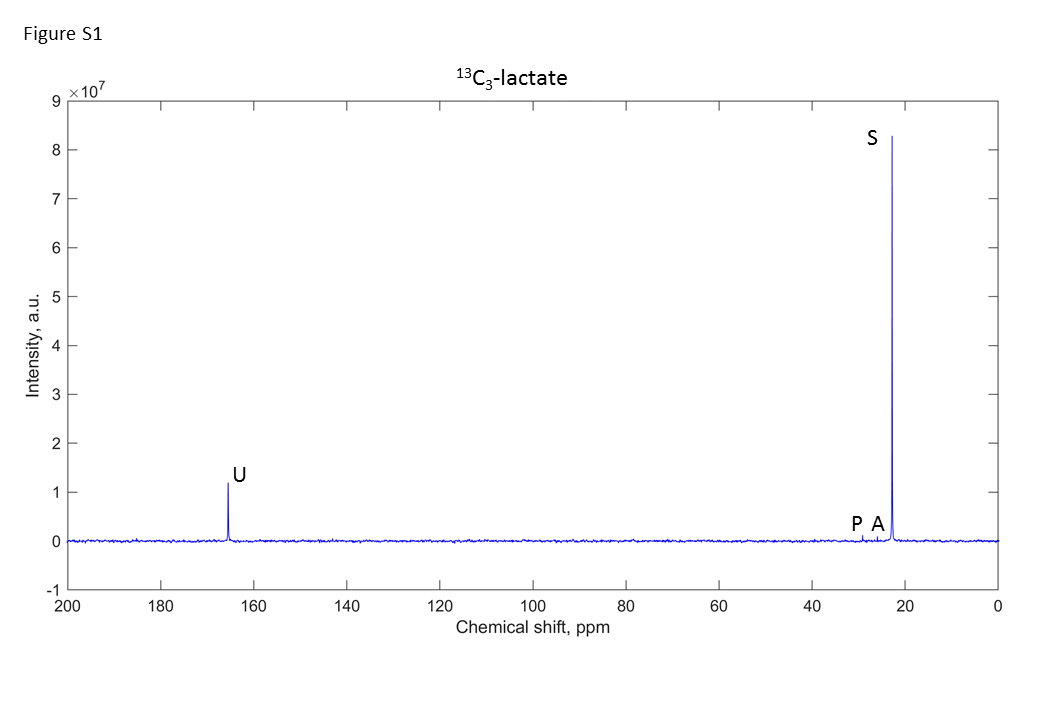

Supplement: Supplementary Data [file gay046_newsuppfig1.png]

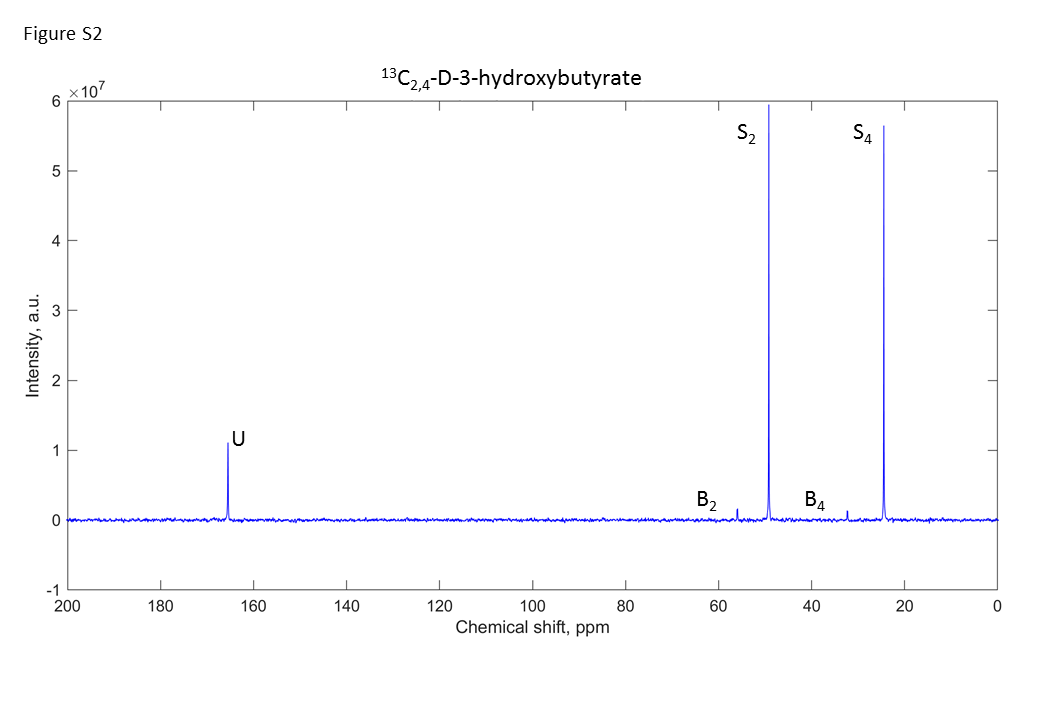

Supplement: Supplementary Data [file gay046_newsuppfig2.png]

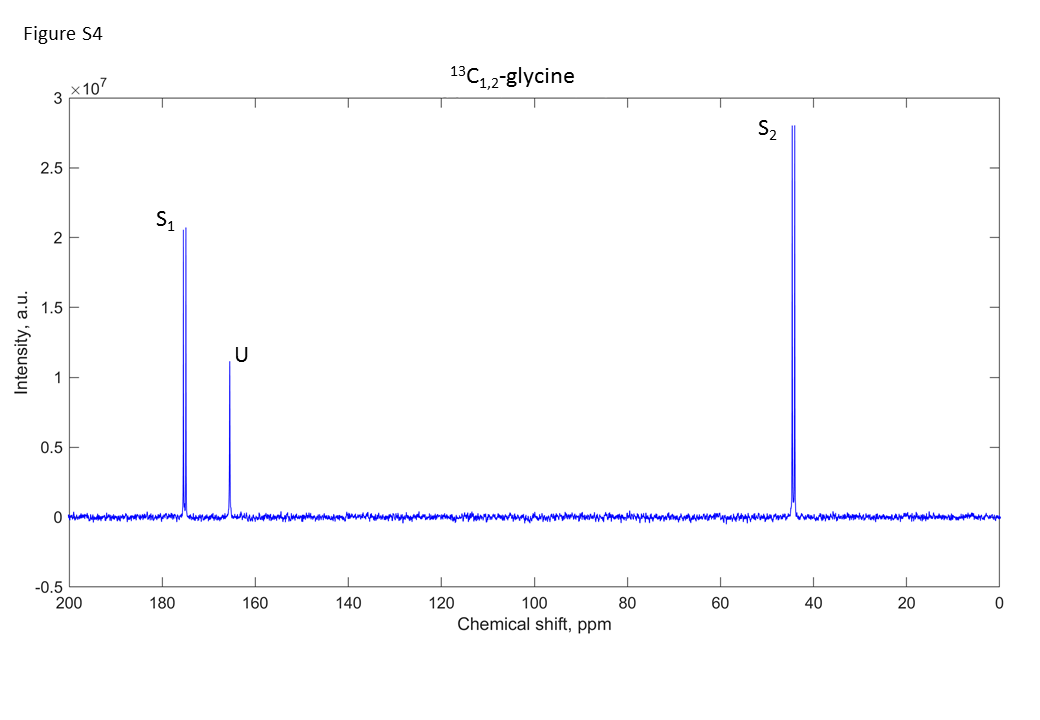

Supplement: Supplementary Data [file gay046_newsuppfig4.png]

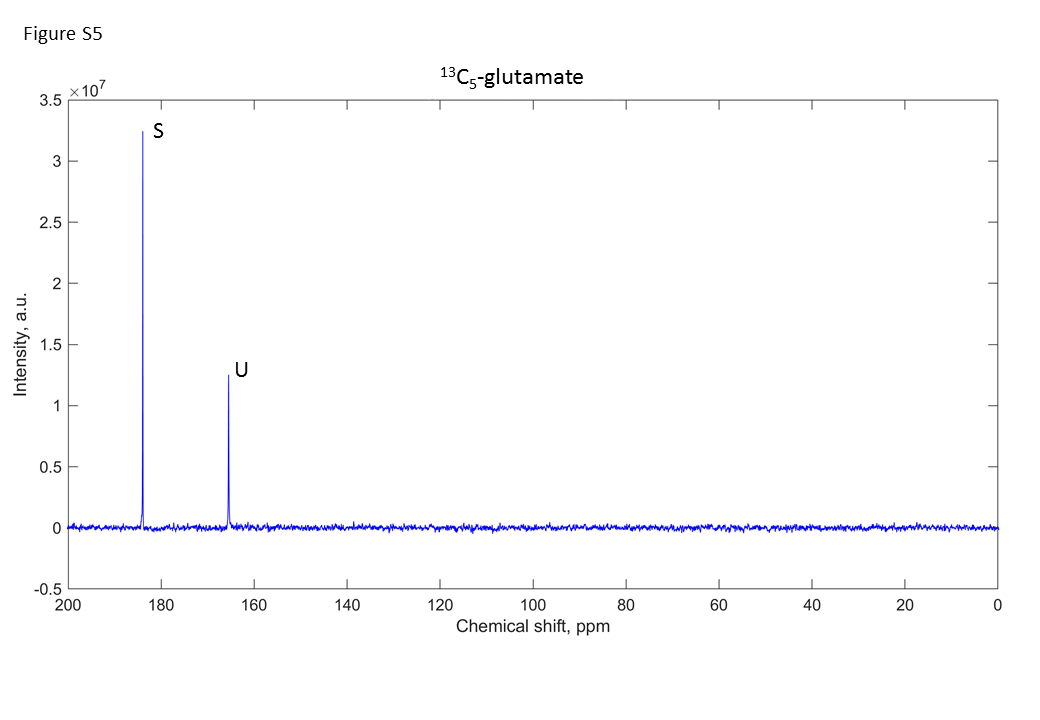

Supplement: Supplementary Data [file gay046_newsuppfig5.png]

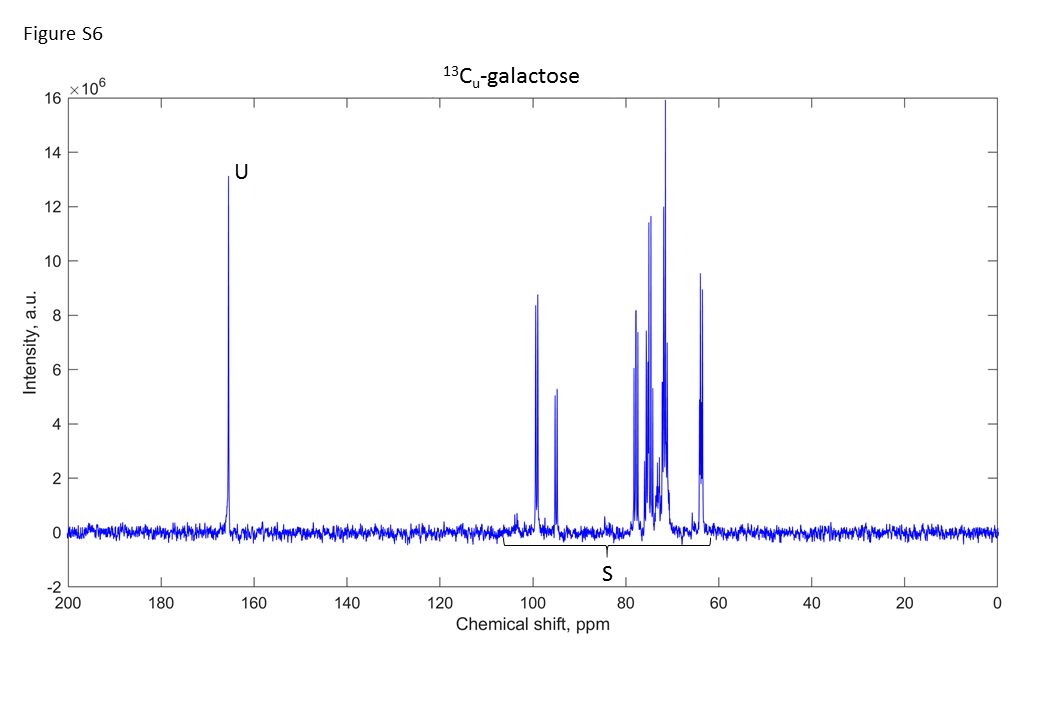

Supplement: Supplementary Data [file gay046_newsuppfig6.png]

## Slide 1
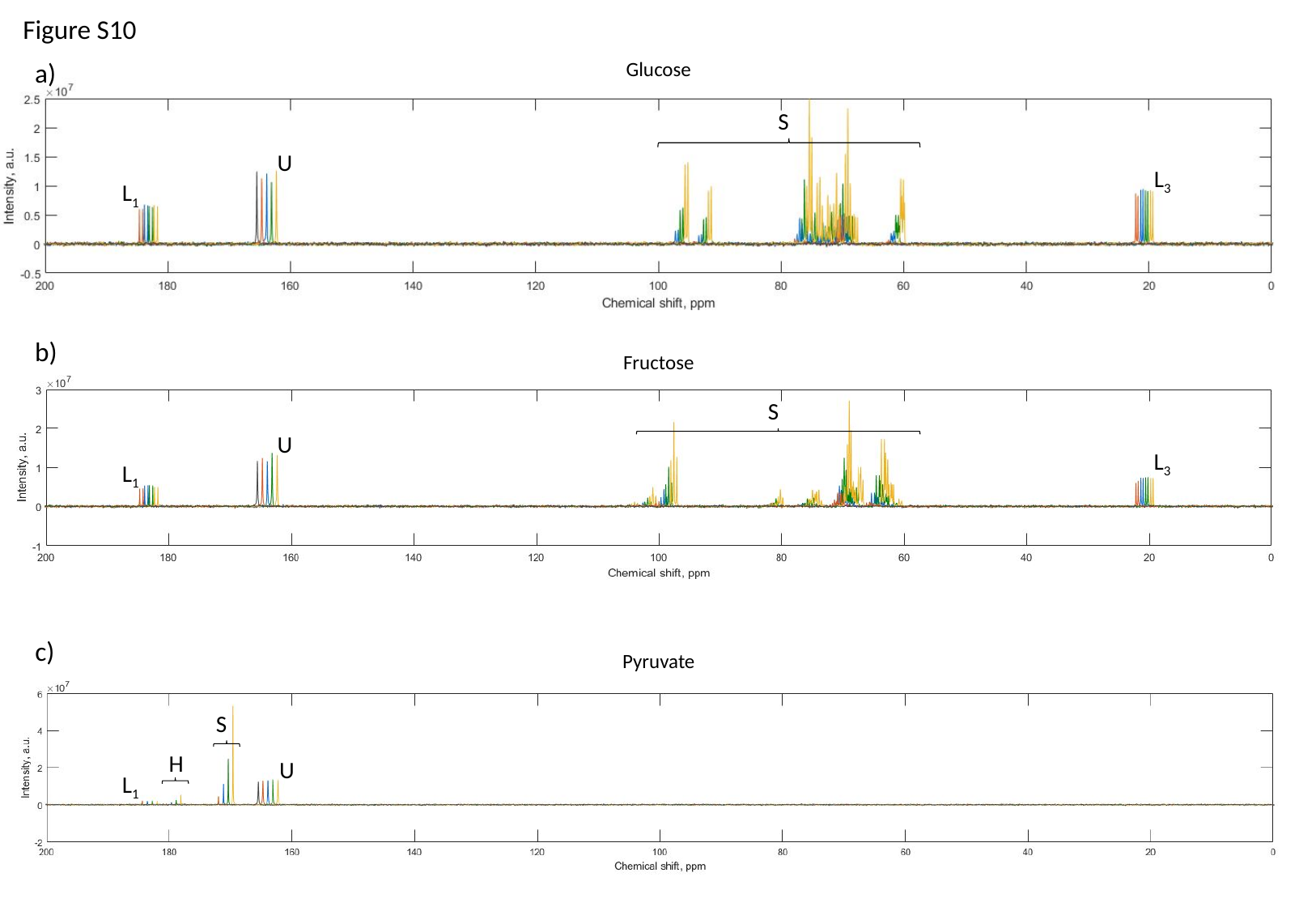

Figure S10
a)
Glucose
S
U
L3
L1
b)
Fructose
S
U
L3
L1
c)
Pyruvate
S
H
U
L1

Supplement: Supplementary Data [file gay046_supplementaryfigure10.pptx]

## Slide 1
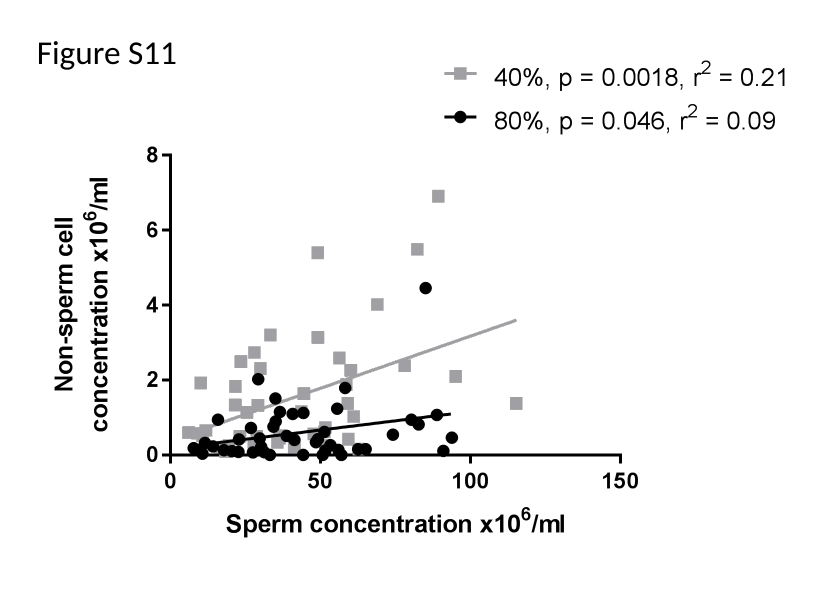

Figure S11

Supplement: Supplementary Data [file gay046_supplementaryfigure11v7.pptx]

## Slide 1
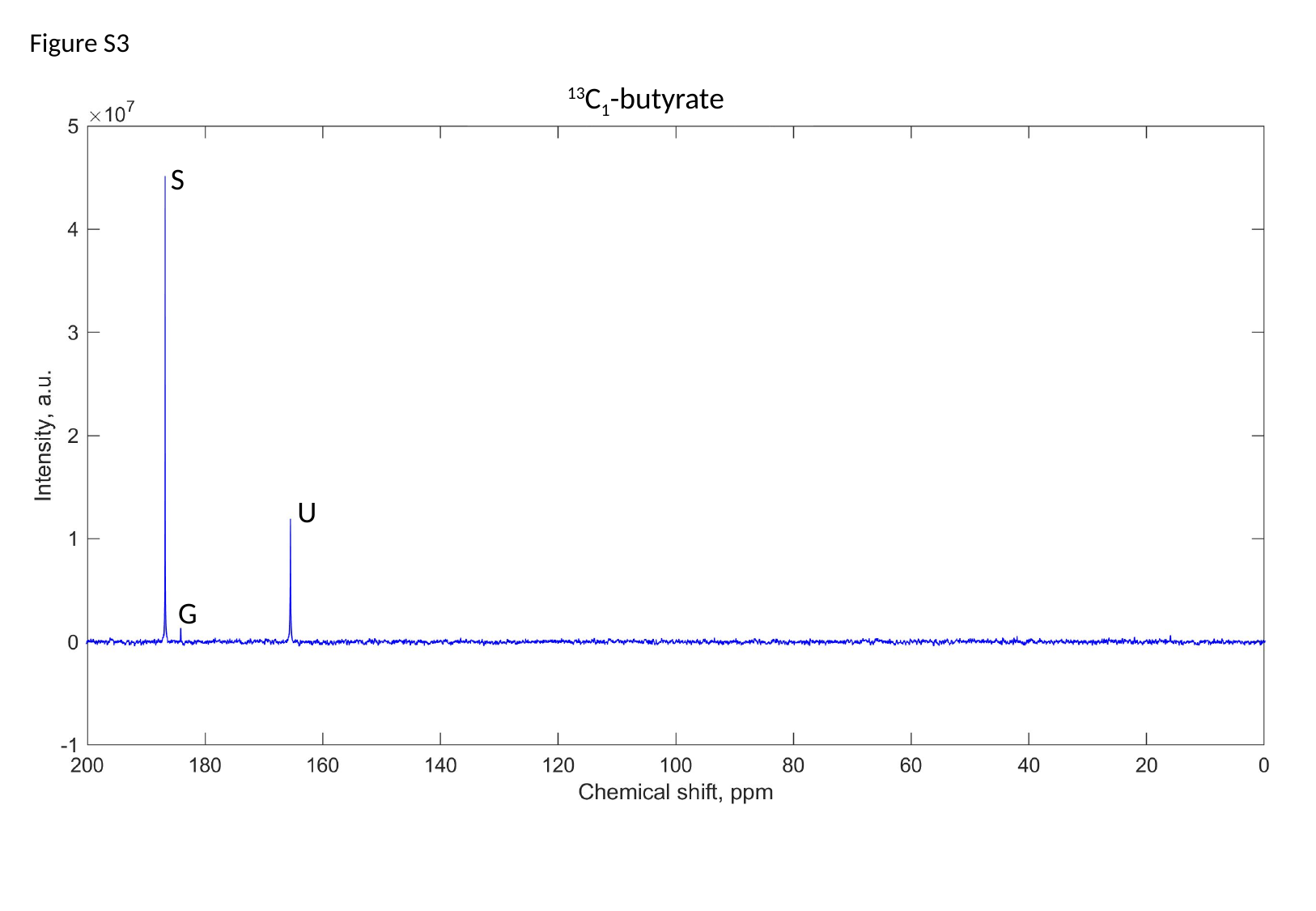

Figure S3
13C1-butyrate
S
U
G

Supplement: Supplementary Data [file gay046_supplementaryfigures3.pptx]

## Slide 1
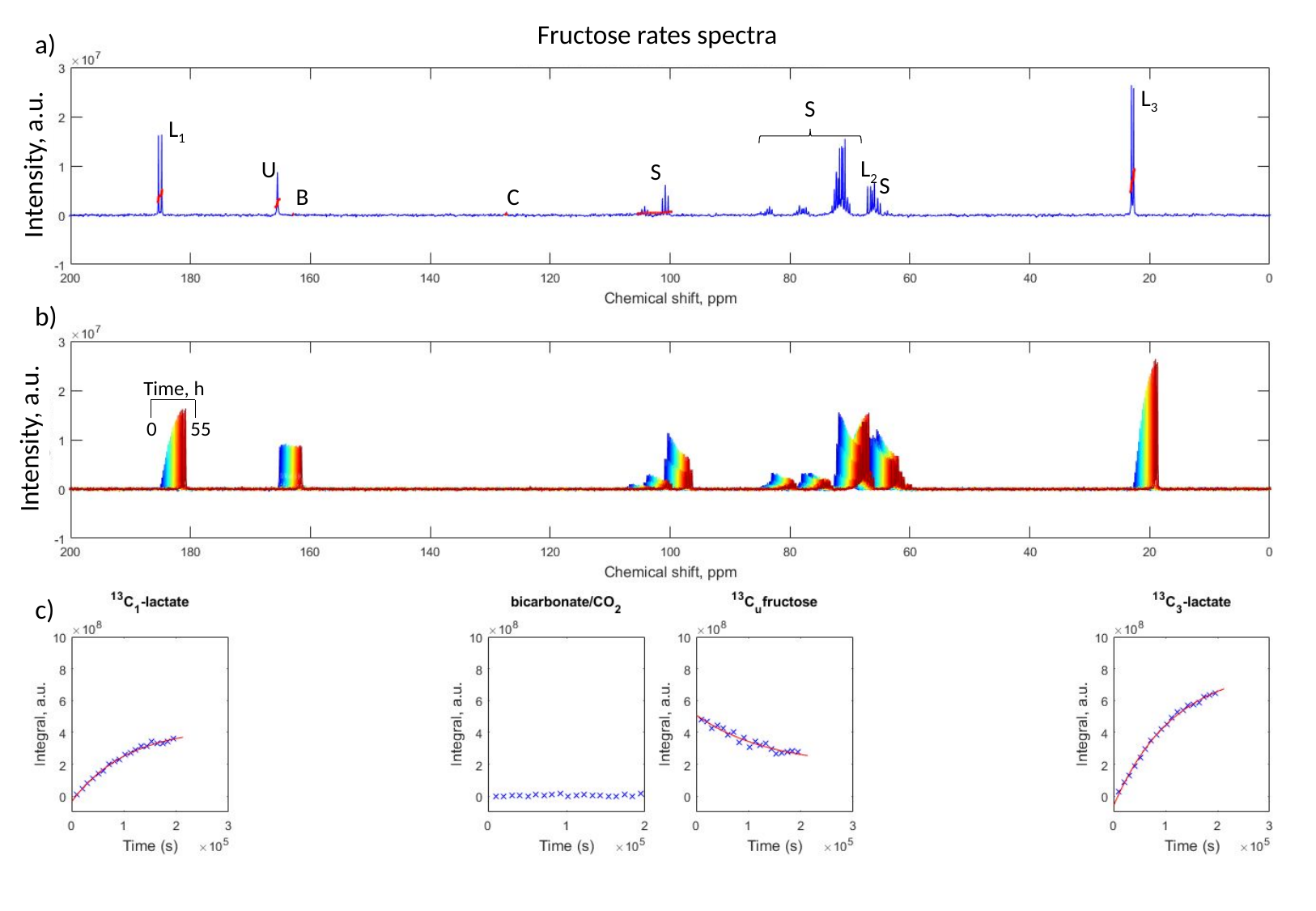

Fructose rates spectra
a)
L3
S
L1
Intensity, a.u.
L2
U
S
S
B
C
b)
Time, h
0
55
Intensity, a.u.
c)

Supplement: Supplementary Data [file gay046_supplementaryfigures7.pptx]

## Slide 1
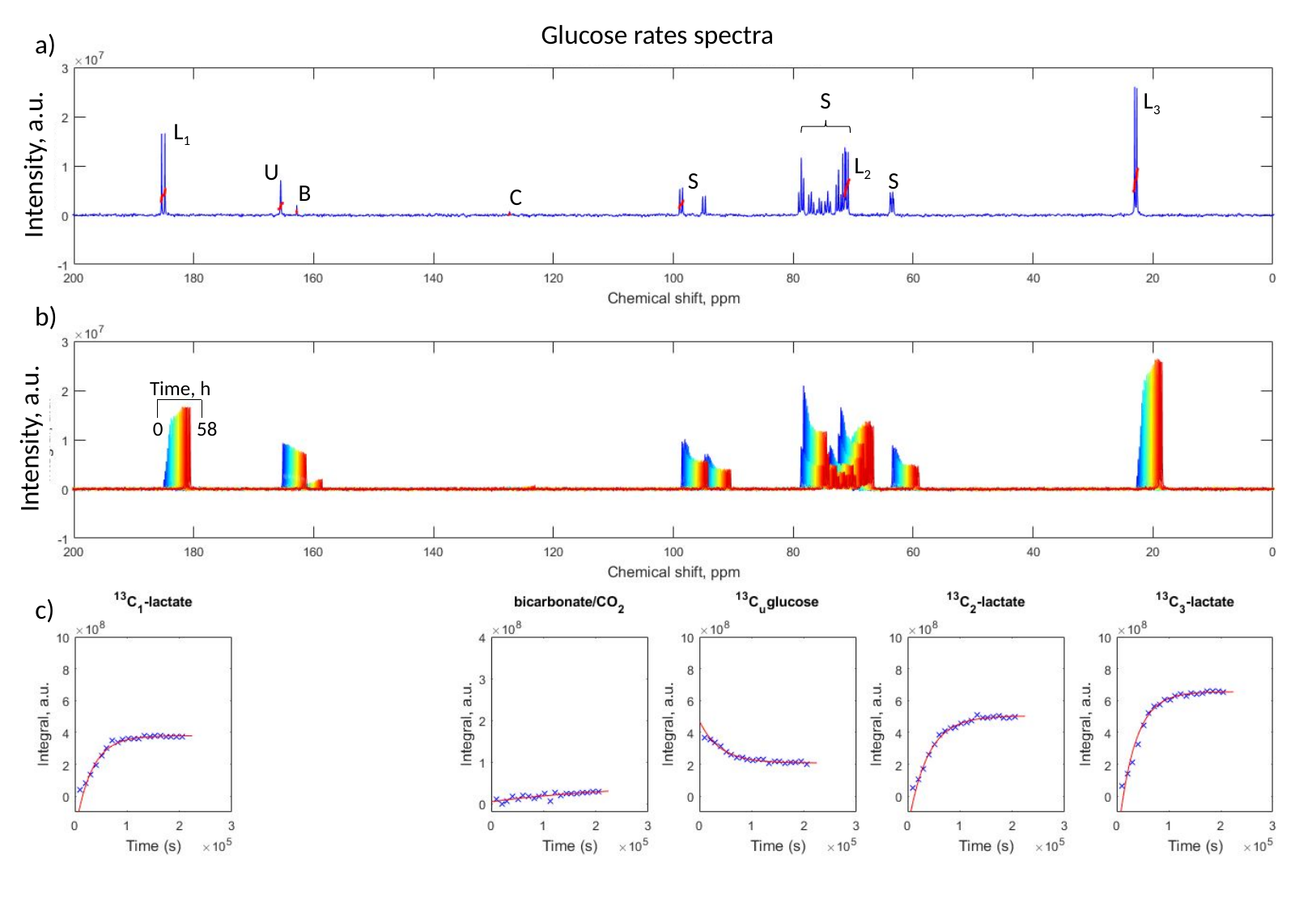

Glucose rates spectra
a)
S
L3
L1
Intensity, a.u.
L2
U
S
S
B
C
b)
Time, h
0
58
Intensity, a.u.
c)

Supplement: Supplementary Data [file gay046_supplementaryfigures8.pptx]

## Slide 1
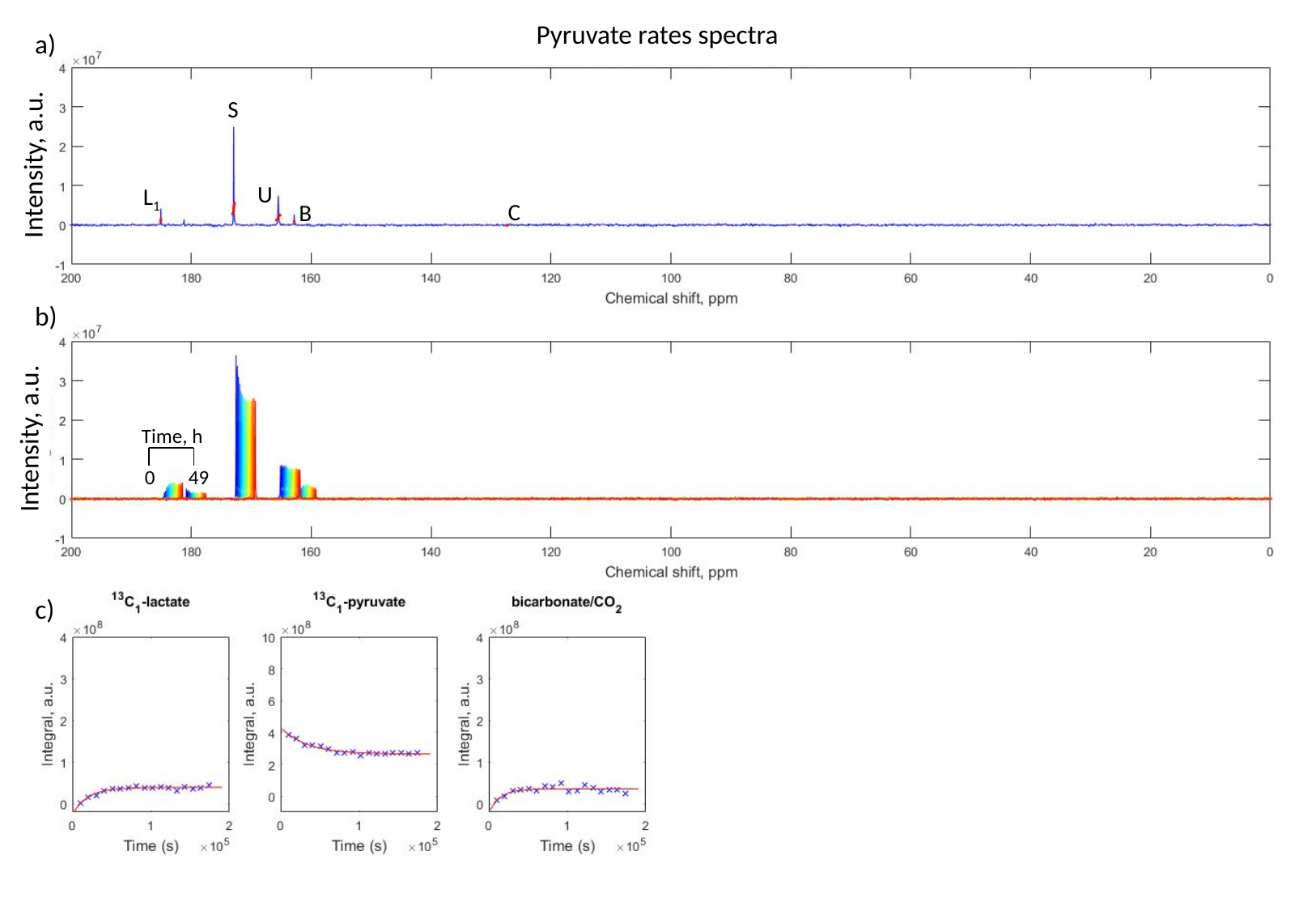

Pyruvate rates spectra
a)
S
Intensity, a.u.
U
L1
C
B
b)
Intensity, a.u.
Time, h
0
49
c)

Supplement: Supplementary Data [file gay046_supplementaryfigures9.pptx]
